# Correlates of mental health in occupations at risk for traumatization: a cross-sectional study

**DOI:** 10.1186/s12888-020-02704-y

**Published:** 2020-06-25

**Authors:** Sarah K. Schäfer, M. Roxanne Sopp, Marlene Staginnus, Johanna Lass-Hennemann, Tanja Michael

**Affiliations:** Department of Psychology, Saarland University, Building A1 3, 66123 Saarbruecken, Germany

**Keywords:** Resilience, Sense of coherence, Salutogenesis, Locus of control, Posttraumatic stress, Burnout, Occupation, Police, Firefighters, Medical staff

## Abstract

**Background:**

Hospitals, police stations, and fire departments are highly demanding workplaces. Staff members are regularly exposed to various stressors including traumatic events. Correspondingly, several studies report high rates of mental health issues among these occupations. Nevertheless, despite these challenging circumstances, some staff members manage to sustain their mental health. The current study is the first to investigate three health-promoting factors simultaneously among three different, highly demanding occupations.

**Methods:**

The present cross-sectional survey investigated health-promoting factors (sense of coherence – SOC, trait-resilience, locus of control – LOC) and mental health outcomes (general psychopathological symptom burden, posttraumatic stress, burnout) in medical staff (*n* = 223), police officers (*n* = 257), and firefighters (*n* = 100).

**Results:**

Among all occupations, SOC, trait-resilience, and an internal LOC were negatively associated with general psychopathological symptoms, posttraumatic stress, and burnout symptoms. By contrast, all these outcome measures were positively correlated with an external LOC. Multiple regression models including all health-promoting factors explained 56% of the variance in general psychopathological symptoms and 27% in posttraumatic stress symptoms. Among all occupations, SOC was the strongest predictor of both general psychopathological symptom burden and posttraumatic stress symptoms. Multigroup path analyses revealed minor differences across occupations, mainly driven by a stronger influence of LOC in police officers.

**Conclusion:**

Across all occupations, SOC was identified as the most important health-promoting factor. Future longitudinal studies should further examine the causal link between health-promoting factors and mental distress in different workplaces. Such studies will also allow for further development and evaluation of resilience promoting programs.

## Background

Some professions are not only exposed to considerable levels of occupational stress but are also at a high risk for experiencing traumatic events. While approximately 70% of the global civilian population report the experience of a traumatic event during their lifetime [1, 2], this statistic increases to 84% for individuals working in high-risk occupations (e.g., police officers, firefighters, and emergency dispatchers [3];). Critically, individuals working in these occupations are repeatedly exposed to work-related traumatic events resulting in a cumulative burden which, in turn, increases their risk of developing mental health problems [4]. Three commonly identified high-risk occupations are medical staff [5], police officers [6, 7], and firefighters [8]. Accordingly, various studies report increased rates of burnout and depression in medical staff (e.g., [9, 10]), especially in intensive care medicine [11]. In case of police officers, symptom severity of mental health problems seem to depend on specific context factors: While a comparative study in the Netherlands did not find increased rates of mental health problems in police officers [12], studies conducted in Austria [13] and Sri Lanka [14] report higher rates of depression among police staff. However, the latter two lack a matched control group of other occupations with lower risks for traumatization and compare the prevalence rates to rates of the general population. Regarding firefighters, reported rates of posttraumatic stress disorder (PTSD) and other mental health problems differ considerably because of various applied cut-off scores and different (mostly self-report) instruments [15]. However, recent findings suggest high rates of mental health issues, including depression, PTSD, as well as substance abuse, and a linear relationship between the number of fatal incidents and the severity of mental health problems [16].

However, responses to occupational and operational stressors vary among employees. While some individuals experience the described mental health problems, others manage to maintain their mental health even when faced with persisting stressful circumstances (e.g., [17–19]). Based on these diverging responses to long-term stressors, it is crucial to identify factors and strategies that enable successful coping in highly demanding workplaces.

In this context, Aaron Antonovsky’s theory of salutogenesis [20, 21] – with sense of coherence (SOC) as its key component – is closely linked to successful coping processes. SOC is defined as ‘a global orientation that expresses the extent to which one has a pervasive and enduring, though dynamic, feeling of confidence that one’s internal and external environments are predictable, and that there is a high probability that things will work out as well as can reasonably be expected’ ([20], p. 10). In line with this definition, SOC as a resistance factor is assumed to uniquely combine behavioural, cognitive, and motivational aspects of coping and resistance [22]. For work stressors, previous studies identify SOC as the most important correlate of mental health problems and posttraumatic stress in intensive care and anaesthesiology staff [23] and paramedics [24]. Moreover, recent meta-analyses underline SOC’s role as a correlate of posttraumatic stress symptoms in various populations [25] and as a determinate of carer well-being in home care settings [26]. Consequently, higher levels of SOC are associated with lower levels of psychopathological symptoms [24] and enhanced posttraumatic growth [27] in medical staff. Similar associations of SOC and mental health problems have also been demonstrated for police officers [28] and firefighters [29].

Another concept considered to be important for maintaining mental health even under stressful circumstances is resilience [30]. However, specific conceptualizations of resilience differ: Firstly, resilience can be defined as a (rather stable) personality trait that inoculates individuals against the negative impact of stressful life events [31]. Secondly, resilience can be conceptualized as an outcome, i.e., as the absence of psychopathological symptoms after loss and potential trauma [32, 33]. Furthermore, a third conceptualisation of resilience as an active process of recovery following aversive life events has been increasingly employed in recent research [34]. Overall, resilience can be broadly defined as the ability to adapt successfully in the face of adversity, trauma, tragedy, or significant threat [35].

When considering resilience as a personality trait, it is plausible to assume that it is involved in the process of coping by enabling an individual to adapt even in challenging situations, thereby contributing to a beneficial outcome in terms of fewer psychopathological symptoms. Trait-resilience is not reflected in a specific coping style and strongly depends on environmental circumstances, i.e., someone can be characterized as *resilient* when his/her behaviour meets environmental demands for successful adaptation (for a review on resilience and its definitions, see Fletcher and Sarkar [36]). Considering related health-promoting variables, trait-resilience shows substantial overlap with the concept of SOC: Both SOC and trait-resilience are assumed to initiate, modulate, and support successful coping processes. However, both concepts have rarely been studied in a joint model with most studies focusing on either SOC or trait-resilience. In this regard, various studies concentrating on trait-resilience have identified associations with fewer psychopathological symptoms in medical staff (e.g., [37–39]), police officers ([40, 41], but see a conflicting study by Balmer, Pooley, and Cohen [42]) as well as in firefighters [43, 44].

Locus of control (LOC, [45]) is another concept that is frequently discussed as a health-promoting factor, which shows substantial conceptual overlap with both SOC and trait-resilience. LOC assesses the degree to which individuals have the impression that events are controllable by their own actions (internal LOC) or predominantly depend on factors beyond their personal influence (external LOC). Previous research has identified an external LOC as a risk factor of posttraumatic stress symptoms [46], as a mediating factor between socioeconomic adversity and later depression [47], and as a correlate of psychopathological symptoms [48]. On the other side, an internal LOC has been demonstrated to be a protective factor against the development of psychopathological symptoms in soldiers [49] and adolescents after an earthquake [46]. In contrast to SOC and trait-resilience, LOC has less frequently been studied across different occupations. However, studies identified LOC as an important correlate of various aspects of mental health in medical staff [50–52], police officers [53–55], and firefighters [56, 57].

As illustrated by the presented evidence, there is a wealth of cross-sectional research on specific health-promoting factors. However, few studies have investigated multiple health-promoting factors simultaneously. Considering their high conceptual overlap, such research is needed to investigate their unique associations with psychopathological symptoms, and to identify the most important predictors and correlates of beneficial health outcomes. While some studies have already considered different concepts and their unique impact on mental health problems [23, 24, 58], to our knowledge, none of these studies simultaneously assessed different high-risk occupations. One cross-sectional study that assessed social resources, including SOC, in multiple uniformed services (i.e., police officers, firefighters, prison officers, security guards, and city guards), focused their analyses around a general model of health and work stress rather than on group comparisons [59]. Given this lack of research, the current study was the first to simultaneously assess multiple health-promoting factors (SOC, trait-resilience, and LOC), as well as psychopathological symptoms (i.e., general mental health problems, posttraumatic stress symptoms, and burnout) in three high-risk occupations. Previous studies assessed only one health-promoting factor among different occupations [29] or different health-promoting factors in one occupation [24]. The aim of the current cross-sectional study was to investigate the associations between health-promoting factors and psychopathological symptoms in different occupations in order to examine their unique contributions to psychopathological symptoms. Critically, we aimed to determine whether different patterns of associations emerge for different occupations by applying multigroup path analyses.

Building on the aforementioned evidence, we hypothesized that all health-promoting factors (except external LOC) would show a significant negative association with mental health outcomes. Moreover, we expected a stronger external LOC to be associated with more severe psychopathological symptoms. Among all health-promoting factors, we hypothesized SOC to be the most relevant predictor of psychopathological symptoms reflected in the largest amount of explained variance in joint regression models [23, 24, 58]. Moreover, we investigated differences in health-promoting factors, psychopathological symptom burden, and patterns of associations for different occupations on an exploratory basis.

## Method

### Sample recruitment

**Fig. 1:**
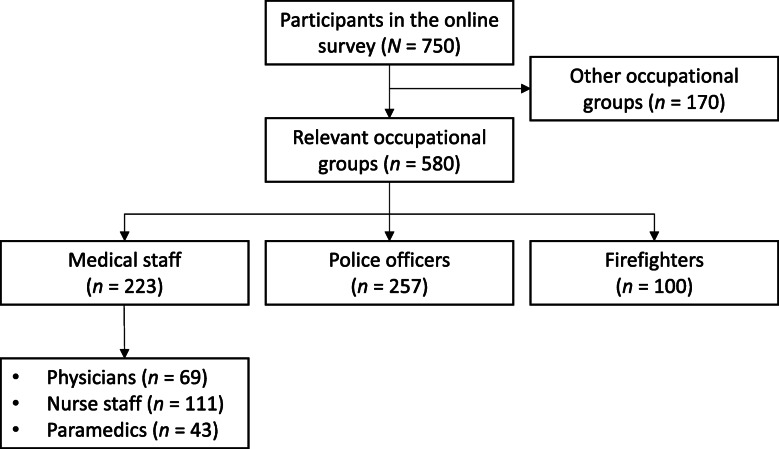
Flow chart of the study sample

Respondents were recruited online by contacting different organisations and interest groups that represent specific high-risk occupations. Specifically, we contacted trade unions for medical professions, police staff, and firefighters. Moreover, study advertisements were posted on webpages addressing members of high-risk occupations (e.g., Facebook groups sharing information on emergency care). Respondents were additionally asked to distribute the survey link at their workplaces. In the case of medical staff, we specifically contacted interest groups and organizations related to fields of medicine that are at high risk for traumatization due to repeated exposure to patient death (i.e., intensive care units, emergency departments, palliative care). Sample recruitment took place between February and November 2018. During this period, 750 individuals completed the 30-min online survey. One hundred seventy respondents were excluded since they did not work in a field of interest (i.e., working in a nursery or an office occupation). The final sample thus comprised 223 respondents who worked in the field of medicine, 257 police officers, and 100 firefighters (see Fig. 1 for a study flow chart). The study protocol was approved by the ethics committee of Saarland University (no. 16–2). All respondents gave written informed consent in accordance with the Declaration of Helsinki [60]. The study sample was also used for a publication on the health-promoting effects of pets [61].

### Sample characteristics

Two hundred and thirty-five women (40.52%) and 345 men (59.48%) with a mean age of 38.19 years (*SD* = ±11.55 years) participated in the survey. Across different occupations, the respondents reported 16.68 years (*SD* = ± 11.54 years) of work experience. Sixty percent of respondents worked in shifts, with 50.51% of those working night shifts and 19.82% working standby shifts.

### Measures

#### Socio-demographic and occupational information

The survey started with 18 questions on socio-demographic characteristics (i.e., gender, marital status, etc.) and occupational information (e.g., type of profession, work experience). This was followed by a set of standardized questionnaires on respondents’ current psychopathological symptom burden and health-promoting factors.

#### Health-promoting factors

##### Sense of coherence

SOC was measured using two questionnaires. SOC as defined by Antonovsky [20] was assessed using the German 13-item short version of the Antonovsky scales (SOC-13 [62]; English original scale: [63]). SOC-13 uses a bipolar seven-point scale with a verbal anchor on each side. Additionally, SOC-Revised (SOC-R) was assessed using a 13-item questionnaire developed by Bachem and Maercker [64]. In the current sample, SOC-13 showed good internal consistency reflected in a Cronbach’s alpha (*α*) of .84. All analyses presented in this publication are based on the Antonovsky scales [20] and use total scores. Results of analyses focusing on SOC-R will be reported elsewhere.

##### Trait-resilience

The Resilience Scale 11 (RS-11 [65]; English original scale: [66]) assesses general psychological resilience as a trait that enables an individual to cope with stressful life events. RS-11 was developed as a unidimensional short version of the 25-item resilience scale and has been validated in a representative German sample [65]. All items are rated on a bipolar seven-point scale. In the current study, its reliability was good with *α* = .90. All analyses use the RS-11 total score.

##### Locus of control

The concept of locus of control was assessed using the four-item brief scale for the assessment of control beliefs (IE-4 [67]). This instrument consists of two subscales comprising two items each measuring perceived internal and external control. All items are rated on a five-point scale. As expected, items of each scale were correlated, *r*_*internal*_ = .36 and *r*_*external*_ = .37, and both scales were negatively correlated, *r* = −.44. Since there is no IE-4 total score, internal and external dimensions of LOC were analysed separately.

#### Psychopathological symptom burden

##### General psychopathological symptoms

General psychopathological symptom burden was assessed using the German version of the Brief Symptom Inventory (BSI [68]; English original: [69]). The BSI is a 53-item self-report instrument that measures symptomatic distress using nine subscales. For this study, the global severity index (GSI) which indicates general psychopathological symptom burden was used for all analyses. In the current study, the GSI showed good reliability as reflected in *α* = .96.

##### Posttraumatic stress symptoms

Posttraumatic stress was measured using the German version of the Impact of Event Scale-Revised (IES-R [70]; English original scale: [71]). The IES-R assesses symptoms of intrusive re-experiencing, hyperarousal, and avoidance. The questionnaire consists of 22 items each rated on a four-point scale. Item scores are transformed into a non-equidistant format (0, 1, 3, 5) resulting in a minimum total score of 0 and a maximum total score of 110. In line with previous findings [70], the IES-R showed good internal consistency in the current sample for the total score (*α* = .93). All analyses were based on the IES-R total score.

##### Burnout symptoms

The German version of the Maslach Burnout Inventory - General Survey (MBI [72]; English original scale: [73]) was used to assess burnout symptoms in different occupations. The MBI consists of 22 items assessing three domains of burnout: emotional exhaustion (EE), depersonalization (DP), and personal accomplishment (PA). All items are rated on a seven-point scale. Psychometric properties of the scale are sufficient [74] and were also satisfactory in the current sample reflected in high internal consistencies for all subscales (*α*_EE_ = .90, *α*_DP_ = .75, *α*_PA_ = .75). Since there is no MBI total score, analyses were conducted for the separate domains of burnout.

### Data collection and analyses

All measures were collected using the online survey platform SoSci Survey [75]. Analyses were conducted using SPSS version 25 [76], RStudio [77], and the *lavaan* package [78]. Descriptive statistics were computed to illustrate sample characteristics in terms of frequencies, means (*M*), and standard deviations (*SD*) of the variables. To assess differences between different occupations, MANOVAs, and *t*-tests for independent samples were conducted. Bonferroni-Holm’s correction [79] was applied to control for the effects of multiple testing when no hypotheses were specified. Moreover, whenever possible we considered total scores instead of subscale scores to further reduce the effect of multiple testing. Pearson’s bivariate correlation coefficients were used to assess the relationship between SOC, trait-resilience, LOC, and psychopathological symptom burden. Multiple regressions were conducted to determine the unique variance explained by each predictor variable that showed a significant bivariate correlation with the respective outcome variable. To assess the specific relevance of each predictor, multiple hierarchical regressions were conducted including each variable in the last step. The change in *R*^2^ (*∆R*^2^) represents the unique amount of variance accounted for by each predictor. *∆F* was used to assess the significance of *∆R*^*2*^. Due to missing data, degrees of freedom vary between analyses. Path analyses were conducted to compare multiple regression models among different occupations. Regression models were calculated as saturated models (*df* = 0) allowing for varying path coefficients across occupations and were compared with a model constraining all regression coefficients across occupations to be equal. Differences in model fit were assessed using *∆χ*^*2*^-tests. A significant ∆*χ*^*2*^-test indicates significant group differences between the regression model. In these cases, further model tests were conducted to identify paths that varied significantly across occupations. Significant differences between regression coefficients were tested using *z*-tests as previously done by Arble, Daugherty, and Arnetz [80].

## Results

### Demographic group differences

Sample characteristics of each occupation are presented in Table 1. Occupations differed regarding the proportion of women, *χ*^*2*^(2) =129.88, *p* < .001. Police officers and firefighters included predominately male participants whereas the medical staff group comprised more women. Occupational groups also differed in mean age, *F*(2, 574) = 6.37, *p* = .002, *η*^*2*^ = .02. After applying Bonferroni-Holm’s correction, post-hoc tests revealed that police officers were significantly older than medical staff, *t*(457) = − 2.84, *p*_adjusted_ = .010, *d* = 0.27, and firefighters, *t*(345) = 3.06, *p*_adjusted_ = .006, *d* = 0.33. There was no difference between medical staff and firefighters, *t*(319) = 0.79, *p* = .431, *d* = 0.09. Moreover, occupations differed significantly regarding their years of work experience, *F*(2, 574) = 25.42, *p* < .001, *η*^*2*^ = .09. Post-hoc tests revealed that medical staff reported significantly fewer years of work experience than police officers and firefighters, *t*(543) =-6.06, *p*_adjusted_ < .001, *d* = 0.52. However, there was no difference between police officers and firefighters, *t*(543) = 1.93, *p* = .054, *d* = 0.17. Shift work was more common in medical staff and police officers than in firefighters, *χ*^*2*^(2) = 60.11, *p* < .001. Of those working shifts, especially police officers reported a higher number of night shifts, *χ*^*2*^(2) = 23.26, *p* < .001. Standby shifts were most frequent in medical staff compared to lower rates in police officers and firefighters, *χ*^*2*^(2) = 38.94, *p* < .001.

**Table 1:** Sample characteristics per occupational group

	Medical staff	Police officers	Firefighters		*p*
Sex (% women)	68.61	28.40	9.00	*χ*^*2*^(2) =129.88	< .001
Age (in years)	37.05 (11.64)	40.05 (11.35)	35.96 (11.26)	*F*(2, 574) = 6.37	.002
Job experience (in years)	12.34 (9.69)	19.82 (11.98)	17.29 (11.16)	*F*(2, 574) = 25.42	< .001
Shift work (%)	74.00	64.20	26.00	*χ*^*2*^(2) = 60.11	< .001
Night shifts (% of those working shifts)	76.43	93.93	69.20	*χ*^*2*^(2) = 23.26	< .001
Standby duty (% of those working shifts)	49.68	16.70	34.62	*χ*^*2*^(2) = 38.94	< .001

Brackets contain standard deviations or degrees of freedom

**Table 2:** Means, standard deviations and group differences of psychopathological symptom burden and health-promoting factors

	Medical staff (MS)	Police officers (PO)	Firefighters (FF)		*p*	Significant post-hoc tests
*Psychopathological symptom burden*						
General psychopathological symptoms (*n* = 571)	15.37 (5.41)	15.91 (5.29)	15.24 (6.38)	*F*(2, 568) = 0.79	.455	
Posttraumatic stress symptoms (*n* = 498)	29.67 (22.49)	30.31 (23.36)	24.58 (19.29)	*F*(2, 495) = 2.31	.101	
*Burnout*						
Emotional exhaustion (*n* = 576)	16.54 (10.35)	18.99 (11.17)	12.01 (10.10)	*F*(2, 573) = 15.26	**< .001**	PO > MS > FF
Depersonalization (*n* = 577)	6.68 (5.95)	9.36 (6.44)	6.57 (5.88)	*F*(2, 574) = 13.80	**< .001**	PO > (MS = FF)
Personal accomplishment (*n* = 572)	30.21 (7.69)	28.06 (8.51)	27.77 (7.93)	*F*(2, 569) = 5.15	**.006**	MS > (PO = FF)
*Health-promoting factors*						
Sense of coherence (*n* = 580)	46.58 (7.59)	45.11 (7.52)	46.84 (7.84)	*F*(2, 577) = 3.02	**.050**	PO < (MS = FF)
Trait-resilience (*n* = 578)	60.94 (10.14)	60.98 (10.18)	60.02 (9.69)	*F*(2, 575) = 0.36	.700	
Internal LOC (*n* = 580)	4.14 (0.62)	3.94 (0.72)	4.18 (0.61)	*F*(2, 577) = 7.05	**.001**	PO < (MS = FF)
External LOC (*n* = 580)	2.40 (0.77)	2.61 (0.82)	2.34 (0.82)	*F*(2, 577) = 5.61	**.004**	PO > (MS = FF)

*Note*. (Marginally) significant group differences are bold. *n*s indicate responses per outcome

*FF* firefighters; *LOC* Locus of control; *MS* Medical staff; *PO* police officers

### Group differences: psychopathological symptom burden

#### General psychopathological symptoms

An ANOVA with occupation as between-subject factor and GSI scores as dependent variable showed no significant group differences regarding psychopathological symptom burden, *F*(2, 568) = 0.79, *p* = .455, *η*^2^ = .00.

#### Posttraumatic-stress symptoms

An ANOVA with occupation as between-subject factor and IES-R total scores as dependent variable revealed no significant group differences, *F*(2, 495) = 2.31, *p* = .101, *η*^2^ = .01.

#### Burnout symptoms

A MANOVA with occupation as between-subject factor and MBI-subscale scores as dependent variables revealed significant group differences, *F*(6, 1134) = 9.89, *p* < .001, *η*^2^ = .05. Univariate comparisons, yielded significant differences for each subscale (see Table 2); emotional exhaustion: *F*(2, 573) = 15.26, *p*_adjusted_ < .001 *η*^2^ = .05; depersonalization: *F*(2, 574) = 13.80, *p*_adjusted_ < .001, *η*^2^ = .05; and personal accomplishment: *F*(2, 569) = 5.15, *p* = .006, *η*^2^ = .02. Post-hoc tests revealed that police officers reported higher levels of emotional exhaustion than medical staff, *t*(573) = 5.06, *p*_adjusted_ < .001, *d* = 0.42, and that emotional exhaustion was higher among medical staff than in firefighters, *t*(573) =-3.50, *p*_adjusted_ < .001, *d* =-0.29. Moreover, police officers showed significantly higher rates of depersonalization compared to both other groups, *t*(574) = 5.10, *p*_adjusted_ < .001, *d* = 0.43, while medical staff and firefighters did not differ, *t*(574) = − 0.14, *p* = .887, *d* =-0.01. Concerning personal accomplishment, medical staff showed higher rates than both other groups, *t*(569) = 3.14, *p*_adjusted_ = .004, *d* = 0.26, while police officers and firefighters reported comparable levels, *t*(569) = 0.30, *p* = .765, *d* = 0.03.

### Group differences: health-promoting factors

#### Sense of coherence

An ANOVA with occupation as between-subject factor and SOC scores as dependent variable revealed marginally significant between-group differences, *F*(2, 577) = 3.02, *p* = .050, *η*^2^ = .010 (see Table 2). Compared to both other groups, police officers showed significantly lower SOC levels, *t*(577) =-2.43, *p*_adjusted_ = .030, *d* =-0.20, while medical staff and firefighters were comparable in SOC levels, *t*(577) =-0.29, *p* = .775, *d* =-0.02.

#### Trait-resilience

In an ANOVA with occupation as between-subject factor and trait-resilience levels as dependent variable, no group differences were found, *F*(2, 575) = 0.36, *p* = .700, *η*^2^ = .00.

#### Locus of control

A MANOVA with occupation as between-subject factor and internal and external LOC scores as dependent variables revealed significant group differences, *F*(4, 1154) = 4.38, *p* = .002, *η*^2^ = .02. Univariate comparisons showed that police officers reported significantly lower internal control beliefs, *t*(577) =-3.72, *p*_adjusted_ < .001, *d* =-0.31, whereas medical staff and firefighters did not differ significantly, *t*(577) =-0.05, *p* = .611, *d* = 0.00. Correspondingly, external control beliefs were significantly higher in police officers, *t*(577) = 3.34, *p*_adjusted_ = .002, *d* = 0.28, while both other groups did not differ, *t*(577) = 0.58, *p* = .560, *d* = .05.

### Bivariate correlations

Table 3 shows the bivariate correlations between health-promoting factors and different measures of psychopathological symptom burden. All health-promoting factors were significantly correlated with mental health outcomes (all *ps* < .001). The strongest association was found between SOC and general psychopathological symptom burden, *r* = −.73, *p* < .001, indicating that a stronger SOC was related to lower symptom levels. As hypothesized, higher levels of SOC, trait-resilience, and a stronger internal LOC were related to less severe general psychopathological symptoms, lower levels of posttraumatic stress, and fewer burnout symptoms. Conversely, stronger external control beliefs were linked to more severe psychopathological symptoms, higher levels of posttraumatic stress, and more burnout symptoms.

**Table 3:** Bivariate Pearson correlations of health-promoting factors and psychopathological symptoms

	1	2	3	4	5	6	7	8	9
SOC (1)	*.84*	.54**	.50**	−.53**	−.73**	−.49**	−.59**	−.44**	.42**
Resilience (2)		*.90*	.45**	−.31**	−.52**	−.34**	−.40**	−.23**	.48**
LOC_internal_ (3)			*.36*	−.44**	−.38**	−.35**	−.42**	−.24**	.33**
LOC_external_ (4)				*.37*	.43**	.38**	.41**	.24**	−.18**
GSI (5)					*.96*	.53**	.59**	.37**	−.32**
IES-R_total_ (6)						*.93*	.45**	.27**	−.30**
MBI_EE_ (7)							*.90*	.58**	−.25**
MBI_DP_ (8)								*.75*	−.20**
MBI_PA_ (9)									*.75*

*Note*. The diagonal shows the reliabilities (Cronbach’s *α*)

** *p* < .001

*SOC* Sense of coherence; *LOC* Locus of control; *GSI* Global Severity Index as measured by the Brief Symptom Inventory to indicate general psychopathological symptom burden; *IES-R* Impact of Event Scale-Revised to assess PTSD symptoms; *MBI* Maslach Burnout Inventory; *MBI*_*EE*_ MBI Emotional exhaustion; *MBI*_*DP*_ MBI Depersonalization; *MBI*_*PA*_ MBI Personal accomplishment

### Regression models

#### General psychopathological symptoms

A multiple regression showed that 56% of general psychopathological symptom burden were explained by SOC, trait-resilience, and internal and external control beliefs, *F*(4, 566) = 179.30, *p* < .001. All predictors except for internal control beliefs, *β* = .05, *t*(566) = 1.33, *∆R*^2^ = .00, *p* = .186, accounted for a unique amount of variance in symptom severity [SOC: *β* = −.61, *t*(566) =-16.10, *∆R*^2^ = .20, *p* < .001; trait-resilience: *β* = −.19, *t*(566) =-5.57, *∆R*^2^ = .02, *p* < .001; external control beliefs: *β* = .07, *t*(566) = 2.16, *∆ R*^2^ = .00, *p* = .031].

#### Posttraumatic-stress symptoms

Regarding posttraumatic stress symptoms, 27% of variance in symptom severity could be collectively explained by the set of health-promoting factors, *F*(4, 493) = 45.18, *p* < .001. However, only SOC, *β* = −.33, *t*(493) = -6.13, *∆R*^2^ = .06, *p* < .001, and an external LOC, *β* = .15, *t*(493) = 3.20, *∆R*^2^ = .02, *p* < .001, accounted for unique amounts of variance.

#### Burnout symptoms

Together, SOC, trait-resilience, and LOC explained 38% of the variance of symptoms of emotional exhaustion, *F*(4, 571) = 88.19, *p* < .001. On a single predictor level, all variables were significant predictors of emotional exhaustion, with SOC being the strongest, *β* = −.43, *t*(571) =-9.63, *∆R*^2^ = .10, *p* < .001, followed by internal LOC, *β* = −.12, *t*(571) =-2.98, *∆R*^2^ = .01, *p* = .003, external LOC, *β* = .10, *t*(571) = 2.58, *∆R*^2^ = .01, *p* = .010, and trait-resilience, *β* = −.09, *t*(571) =-2.16, *∆R*^*2*^ = .01, *p* = .031. Regarding depersonalization, only 19% of the variance were explained by all predictors, *F*(4, 572) = 33.70, *p* < .001, whilst only SOC accounted for an unique amount of variance, *β* = −.42, *t*(572) =-8.32, *∆R*^2^ = .10, *p* < .001. Concerning personal accomplishment, the set of predictors accounted for 28% of the variance, *F*(4, 567) = 53.79, *p* < .001. Trait-resilience was the strongest predictor, *β* = .34, *t*(567) = 7.84, *∆R*^*2*^ = .08, *p* < .001, followed by SOC, *β* = .23, *t*(567) = 4.70, *∆R*^2^ = .03, *p* < .001, an internal LOC, *β* = .10, *t*(567) = 2.27, *∆R*^2^ = .01, *p* = .024, and an external LOC, *β* = .09, *t*(567) = 2.01, *∆R*^2^ = .01, *p* = .045. See Additional File 1 for a table presenting all regression results.

### Group differences: health-promoting factors

#### General psychopathological symptoms

Comparing two models predicting general psychopathological symptom burden based on SOC, trait-resilience, internal, and external LOC allowing the regression coefficients to vary across groups or not, had no impact on the model fit, ∆*χ*^*2*^(8) = 12.91, *p* = .115, indicating no differences between the occupations regarding the prediction of general psychopathological symptom burden.

#### Posttraumatic-stress symptoms

Applying the same model comparison to posttraumatic stress, the test revealed a significant difference between models, ∆*χ*^*2*^(8) = 22.15, *p* < .001. Model comparisons between models fixing regression coefficients across all groups and models allowing one path to vary across groups, revealed significant fit differences for external LOC, ∆ *χ*^*2*^(2) = 9.25, *p* = .001 (see Table 4 for all paths). Regarding regression coefficients, SOC descriptively remained the strongest predictor of posttraumatic stress for all occupations (see Table 5). However, external control beliefs explained a larger amount of variance in posttraumatic stress symptoms in police officers compared to firefighters, *diff* = .31, *p*_adjusted_ < .001, and medical staff, *diff* = .21, *p*_adjusted_ < .001, but there was no difference between medical staff and firefighters, *diff* = .10, *p* = .111, where external control beliefs were no longer a significant predictor of posttraumatic stress symptoms.

**Table 4:** Fit differences between models fixing all regression coefficients across groups and models allowing one path to vary across groups

Outcome	Model comparisons
**Posttraumatic stress**	
Sense of coherence	∆*χ*^*2*^(2) = 5.67, *p* = .059
Trait-resilience	∆*χ*^*2*^(2) = 4.55, *p* = .103
Internal LOC	∆*χ*^*2*^(2) = 2.18, *p* = .337
External LOC	∆*χ*^*2*^(2) = 9.25, *p* = **.001**
**Burnout**	
*Emotional exhaustion*	
Sense of coherence	∆*χ*^*2*^(2) = 1.20, *p* = .548
Trait-resilience	∆*χ*^*2*^(2) = 4.41, *p* = .111
Internal LOC	∆*χ*^*2*^(2) = 2.84, *p* = .242
External LOC	∆*χ*^*2*^(2) = 0.95, *p* = .620
*Personal accomplishment*	
Sense of coherence	∆*χ*^*2*^(2) = 6.34, *p* = **.042**
Trait-resilience	∆*χ*^*2*^(2) = 17.72, *p* **< .001**
Internal LOC	∆*χ*^*2*^(2) = 10.53, *p* = **.005**
External LOC	∆*χ*^*2*^(2) = 10.05, *p* = **.007**

*Note*. Significant group differences are **bold**. *LOC* Locus of control

**Table 5:** Differences of path analyses between occupations

	Medical staff	Police officers	Fire-fighters	|*diff* 1|	*p*_adjusted_	|*diff* 2|	*p*_adjusted_	|*diff* 3|	*p*
**General psychopathological symptoms**									
Sense of coherence	**−.68**	**−.49**	**−.68**						
Trait-resilience	**−.12**	**−.25**	**−.26**						
Internal LOC	.08	.04	.02						
External LOC	.02	**.05**	.05						
**Posttraumatic stress**									
Sense of coherence	**−.24**	**−.36**	**−.44**	.20	.174				
Trait-resilience	.01	**−.15**	.06	.21					
Internal LOC	−.14	.06	**−.25**	.31					
External LOC	.07	**.28**	−.03	.31	*< .001*	.21	*< .001*	.10	.111
**Burnout**									
*Emotional exhaustion*									
Sense of coherence	**−.57**	**−.28**	**−.36**	.29					
Trait-resilience	−.02	**−.25**	−.04	.23					
Internal LOC	−.04	−.10	**−.27**	.23					
External LOC	.02	**.15**	.11	.09					
*Depersonalization*									
Sense of coherence	**−.43**	**−.43**	**−.39**						
Trait-resilience	−.04	−.03	.13						
Internal LOC	.09	−.06	−.10						
External LOC	−.06	−.04	.10						
*Personal accomplishment*									
Sense of coherence	**−.44**	**−.43**	**−.39**	.05	*.021*	.04	.082		
Trait-resilience	−.04	−.03	.13	.17	.100				
Internal LOC	.09	−.06	−.10	.19	.099				
External LOC	−.06	−.04	.09	.15	.840				

*Note*. Unstandardized coefficients are reported as estimated in the grouped path analysis. Significant regression coefficients in each group model are bolded (*p* < .05). Differences between medical staff, police officers, and firefighters are italicized for emphasis. *p*-values are adjusted using Bonferroni-Holm’s correction.

*diff 1* = Largest difference between regression coefficients that could be calculated. *diff* 2 = Second largest difference. *diff* 3 = Remaining comparison. *LOC* Locus of control

#### Burnout symptoms

Concerning burnout symptoms, the model comparison indicated significant differences across the different occupations regarding emotional exhaustion, ∆*χ*^*2*^(8) = 17.40, *p* = .026, and personal accomplishment, ∆*χ*^*2*^(8) = 28.92, *p* < .001, but no differences for depersonalization, ∆*χ*^*2*^(8) = 7.31, *p* = .504. Concerning emotional exhaustion, model comparisons did not reveal significant fit differences for models allowing one path to vary across groups (see Table 4). Regarding personal accomplishment, model comparisons showed significant fit differences between a model fixing all regression coefficients and a model allowing one path to differ across groups for each predictor variable. However, comparing the regression coefficients between the occupations, there was only one significant difference reflected in a larger association of SOC and personal accomplishment in medical staff than in firefighters, *diff* = .05, *p*_adjusted_ = .021.

## Discussion

For the first time, the current study assessed multiple health-promoting factors and their associations with psychopathological symptoms across different high-risk occupations, i.e., medical staff, police officers, and firefighters. SOC was identified as the most important correlate of psychopathological symptoms across different occupations. While all health-promoting factors were found to collectively explain 56% of the variance in general psychopathological symptom burden and 27% of differences in posttraumatic-stress, SOC emerged as the strongest predictor for both outcome variables, uniquely accounting for 20% of variance in general psychopathological symptom burden and 6% in posttraumatic stress symptoms. SOC was also the strongest predictor of the burnout subscales of emotional exhaustion and depersonalization symptoms and explained an equal amount of variance as trait-resilience in personal accomplishment scores. Moreover, path analyses investigating group differences in the regression models did not reveal differences for general psychopathological symptom levels but found significant differences between occupations for posttraumatic stress and burnout symptoms (except for depersonalization).

The current findings are in line with previous research that identified SOC as an important correlate of psychopathological symptoms across different occupations (e.g., [24, 25]). Comparing different health-promoting factors, SOC’s particularly strong association with several mental health outcomes may result from its conceptualization as the most comprehensive resistance factor, uniquely combining cognitive, motivational, and behavioral aspects that are essential in dealing with various stressors [22]. All health-promoting factors investigated in this study share aspects of (internal) control. Moreover, especially trait-resilience and SOC may also have the expectancy of positive outcomes of coping processes in common. However, SOC uniquely assesses the impact of meaning in life (e.g., SOC item: ‘Until now your life has had: No clear goals or purpose at all – Very clear goals and purpose.’) [58]. Thereby, the SOC scale may capture a relevant aspect of spirituality that might be associated with better mental health (see Dein et al. [81] for a critical review). *Meaningfulness* is also one of the subscales assessed by the Antonovsky scales [63], however, due to the questionable factorial validity of these scales [82] and to limit the number of comparisons, we decided to focus our analyses on total scores. However, future studies should further explore SOC’s unique ability to account for variance in relevant outcomes above other health-promoting factors. These studies also need to address the question whether this predictive value of SOC is mainly linked to its assessment using the Antonovsky scales [63] or if SOC’s superiority above other health-promoting factors reflects a more comprehensive concept on a theoretical level.

However, other aspects than SOC might also be of interest: In contrast to previous findings from our group [23, 24], trait-resilience, as well as internal and external control beliefs, also accounted for significant amounts of variance in general psychopathological symptom burden and posttraumatic stress. Nonetheless, in terms of effect sizes, SOC remained the strongest correlate of mental health outcomes. The significant associations with trait-resilience and control beliefs might thus be driven by our large sample size (but see Streb et al. [24] with *N* = 668 paramedics), which also allowed for the identification of smaller predictors. However, despite SOC’s role as an important correlate of mental health, its vague conceptual boundaries have been debated [64]. SOC’s strong correlations with other constructs, including depression, anxiety, and neuroticism, challenge its role as an independent concept [83, 84] as they suggest that SOC might constitute an inverse measure of psychopathology. However, there is no substantial overlap in item content between the SOC scales [63] and standard measures of depression or anxiety. Furthermore, SOC increases over time and is found to be particularly strong in older adults [22, 85], whereas the exact inverse course was not observed for measures of mental health issues [86]. Thus, reducing SOC to an inverse measure of psychopathology seems inappropriate. Irrespective of their overlap with other measures, the SOC scales developed by Antonovsky [63] seem to provide an efficient way of assessing different health-promoting aspects that show a substantial and robust association with various domains of mental health.

Concerning group differences, path analyses did not identify differences between the occupations for general psychopathological symptom burden, which in turn showed the strongest association with the investigated health-promoting factors. In contrast, the predictors accounted for differential amounts of variance between groups for posttraumatic stress. Across all occupations, SOC remained the strongest predictor of posttraumatic stress. Interestingly, within the police group as opposed to medical staff and firefighters, an external LOC was found to be a significant and strong predictor for posttraumatic stress. Coincidentally, police officers reported significantly higher levels of an external LOC and significantly lower levels of internal control beliefs and SOC, suggesting an important role of control beliefs in police officers. In line with these findings, prior studies investigating LOC in police staff reported a positive association of external control beliefs and perceived levels of stress (e.g., [54, 55]). Moreover, a recent cross-sectional study by Arble, Daugherty, and Arnetz [80] investigated approach- and avoidance-based coping strategies in Swedish police officers and other non-military first responders. In line with the current findings, they mainly report similarities in coping processes and well-being across different first responders. However, avoidant coping, which describes strategies to avoid direct considerations of emotions and thoughts as well as triggering stimuli related to stressful events, was particularly relevant in police officers. Such coping strategies showed a stronger association with poor well-being and less posttraumatic growth in police officers than in other first responders. Correspondingly, a recent study reported a positive association of passive coping strategies and PTSD symptoms [87]. The current study identified control beliefs as an important correlate of PTSD symptoms, particularly in police officers. Thus, further studies in different occupations should investigate the relationship between control beliefs and avoidant coping, which may be caused by stronger external and weaker internal control beliefs, and might act as a mediator between control beliefs and psychopathological symptoms as shown previously in firefighters [56]. However, given the cross-sectional nature of both studies, these findings do not address if individuals with low levels of internal and high levels of external control beliefs and avoidant coping strategies tend to choose a career in the police or if specific occupational and operational stressors during police work impact on control beliefs. Furthermore, differences in personality between high-risk occupations, as they have been shown between police officers and firefighters [88], may also impact both the choice of occupation and responses to stressors. As the directionality of this association is of critical relevance for potential interventions targeted at the promotion of protective factors in occupations at risk for mental distress, longitudinal studies are urgently required. Further, these studies should also focus on stressors that are specifically relevant to individual occupations, which might influence the differential relevance of health-promoting factors between these occupations.

While general psychopathological symptom burden and posttraumatic stress clearly showed the strongest association with SOC, burnout symptoms, which have not been addressed in prior studies [23, 24, 59], demonstrated a more diverse pattern of associations across different burnout domains. Depersonalization and emotional exhaustion, which showed the strongest correlations with psychopathological symptoms, were mainly predicted by SOC. However, trait-resilience was the strongest predictor of personal accomplishment. Our findings are in line with prior studies that have already identified strong associations between SOC and burnout especially in medical staff [89–91], between trait-resilience and burnout [37, 92, 93], as well as between control beliefs and burnout [51, 52]. Moreover, as opposed to general psychopathological symptoms and posttraumatic stress, occupations differed regarding burnout symptoms. In line with a previous study that described a distinct pattern of results for police staff [80], this study found medical staff and firefighters to report lower levels of burnout symptoms. Together these findings indicate the presence of particular strain within the police ([94–96], but see: [12]). However, given that the current data constitute the first investigation of burnout symptoms within the context of multiple health-promoting factors across different occupations in a large sample, results should be interpreted with caution. Particularly considering that some studies identified problems with the factorial validity of the MBI scales specifically in heavily burdened populations [97, 98].

### Limitations

The present study has several limitations: Firstly, our findings show that SOC, trait-resilience, and LOC are correlates of psychopathological symptoms. However, no causal conclusions can be drawn from the current study: On the one hand, it is plausible to assume that these factors might play an important role in the development and course of psychopathological symptoms. On the other hand, the results might equally reflect that SOC, trait-resilience, and an internal LOC are impaired by current mental health problems and posttraumatic stress. It is also conceivable that a third variable might underlie the relationship between health-promoting factors and psychopathological symptoms. Thus, only longitudinal studies in large samples will give insight into the causal influence of health-promoting factors on psychopathological symptoms and their development. Such studies may also assess a wider range of health-promoting factors (e.g., openness, dispositional optimism, self-efficacy, and sense of mastery) and include a broader assessment of health including physical aspects.

Secondly, the present study did not assess occupational stressors. As these stressors are assumed to influence both health-promoting factors and levels of psychopathological symptoms, future studies should include respective measures. To assess a large sample size across different occupations, we limited the number of measures to ensure that survey participation was not too time-consuming.

Thirdly, our recruitment approach and sample characteristics and their influence on the findings must be considered. We recruited respondents by contacting different organizations and interest groups that represent specific high-risk occupations. Unfortunately, we were unable to gather information on the precise response rates among these organizations. Thus, our sample consists of volunteers willing to participate in an online survey, which could have biased our findings in different ways. On the one hand, it is plausible to assume that those who experience higher levels of stress are more likely to participate in a study related to stressful workplaces. On the other hand, stressed individuals may also refuse to invest their limited time in survey participation. However, since participation in a survey is voluntary per se it is difficult to avoid such a bias. Furthermore, due to data security concerns, we were unable to ensure that every respondent in the medical group worked in a high-risk occupation at the time of survey completion. Based on available data we can ensure that 68.7% were currently working in a high-risk field while 19.7% were not (e.g., ambulatory care services). It is conceivable that some might have worked in these occupations in their past. For another 12.1% we do not have the precise information that would allow for such a differentiation (e.g., they indicated to work in internal medicine but not specificly in an intensive care unit). However, including and excluding these respondents did not impact on our findings. Moreover, we were unable to conduct gender-specific group analyses due to large differences in gender distributions between occupations. Although our sample was generally large, fewer respondents in the police and firefighter groups were female (e.g., nine women working in fire departments vs. 153 women working in medical occupations). We believe that these differences reflect real differences in gender distributions. Notably, some studies found women and men working in high-risk occupations to be more comparable in psychopathological symptom levels than men and women from unselected samples [99, 100]. Nevertheless, future studies should explore the potential impact of gender on differences between high-risk occupations. Moreover, our sample size per occupational group differed (medical staff: *n* = 223; police officers: *n* = 257; firefighters: *n* = 100). This may have negatively impacted our statistical power to detect group differences, particularly for firefighters. Consequently, the generalizability of our findings may be limited by specific characteristics of the study sample and potential selection bias and require replication in representative samples using more elaborate methods of sample recruitment.

### Future research

The majority of studies on mental health problems in different occupations are cross-sectional in design, limited to specific aspects of health, and investigate only a small set of health-promoting factors [101]. Future research should address these shortcomings by including multiple health-promoting factors to further identify, both their unique association with several health outcomes and their overlapping aspects. Consequently, some of the discussed factors may become subordinate as they might only explain minor proportions of redundant variance. Moreover, such studies should also include posttraumatic growth as an outcome measure since it is associated with both health-promoting factors [27] and psychopathological symptoms [102]. Furthermore, there is a strong need for longitudinal studies in representative samples addressing the predictive value of several health-promoting factors across a longer time. A further shortcoming of current research is that some of the very rare longitudinal studies only assess health-promoting factors after prior exposure to several stressors. This may have already impaired health-promoting factors which might influence their assessment [103, 104]. Thus, future studies should assess individuals at the beginning of their professional careers and include assessments of childhood adversity, which was recently found to impact on coping with occupational stressors in later life [105]. Future large-scale studies should assess health-promoting factors as early as possible and more than twice to identify their causal influence on emerging psychopathological symptom burden. Such studies may also allow for further development and evaluation of resilience promoting programs, which have also shown to be effective in non-clinical samples [106].

## Conclusions

The current study is the first to simultaneously address the association of psychopathological symptoms and multiple health-promoting factors across different high-risk occupations (medical staff, police officers, and firefighters). Across all occupations, sense of coherence was the strongest correlate of general psychopathological symptom burden, posttraumatic stress, and burnout. Furthermore, burnout symptoms were strongly correlated with trait-resilience. Overall, the predictors of mental health problems were similar across occupations. However, in contrast to medical staff and firefighters, external control beliefs explained a unique amount of variance in police officers in both general psychopathological symptoms and posttraumatic stress suggesting an important role of control beliefs in police staff. Future studies need to further examine these differences among occupations in representative samples over a longer period of time.

## Supplementary information

**Additional file 1.** Tables presenting regression results.

## Data Availability

The datasets used and/or analysed during the current study are available from the corresponding author on reasonable request.

## Abbreviations

BSI: Brief Symptom Inventory
FF: Firefighters
GSI: Global Severity Index as measured by the Brief Symptom Inventory
IES-R: Impact of Event Scale-Revised
IE-4: A 4-item brief scale for the assessment of control beliefs
LOC: Locus of control
MBI: Maslach Burnout Inventory
MBI_DP_: MBI Depersonalization
MBI_EE_: MBI Emotional exhaustion
MBI_PA_: Personal accomplishment
MS: Medical staff
PO: Police officers
PTSD: Posttraumatic stress disorder
SOC: Sense of coherence
SOC-R: SOC-Revised
RS-11: Resilience Scale 11
