# Supplementary material for: Correlates of mental health in occupations at risk for traumatization: a cross-sectional study
Source: BMC Psychiatry. 2020 Jun 25;20:335. doi: 10.1186/s12888-020-02704-y (PMC7318450; doi:10.1186/s12888-020-02704-y)
Supplement: Supplementary file 1 — Additional file 1. Tables presenting regression results. [file 12888_2020_2704_MOESM1_ESM.pdf]

Additional file 1: Correlates of mental health across occupations: a cross-sectional study

Tables presenting regression results

1. General psychopathological symptom burden

	B	SE *B*	*β*	*t*	*p*	ΔR^2	ΔF
Sense of coherence	-0.44	0.03	-.61	-16.10	< .001**	.20	261.16
Resilience	-0.10	0.02	-.19	-5.57	< .001**	.02	30.41
Locus of control - internal	0.37	0.28	.05	1.32	.186	.00	1.81
Locus of control - external	0.50	.23	.07	2.16	.031*	.00	4.83

Note. * $p < .05$; ** $p < .01$. The columns reporting ΔR^2 and ΔF refer to hierarchical regression analyses in which each variable was included in the last step. *p*-values of the beta-weights and ΔF are equal and hence not reported twice.

2. Posttraumatic stress symptoms

	B	SE *B*	*β*	*t*	*p*	ΔR^2	ΔF
Sense of coherence	-0.97	0.16	-.33	-6.13	< .001**	.06	37.60
Resilience	-0.18	0.11	-.08	-1.65	.100	.00	2.71
Locus of control - internal	-2.19	1.58	-.07	-1.39	.167	.00	1.92
Locus of control - external	4.13	1.29	.15	3.20	.001**	.02	10.26

Note. * $p < .05$; ** $p < .01$. The columns reporting ΔR^2 and ΔF refer to hierarchical regression analyses in which each variable was included in the last step. *p*-values of the beta-weights and ΔF are equal and hence not reported twice.

3. Burnout symptoms

	B	SE *B*	*β*	*t*	*p*	ΔR^2	ΔF
**Emotional exhaustion**							
Sense of coherence	-.062	0.06	-.43	-9.63	< .001**	.10	93.56
Resilience	-0.09	0.04	-.09	-2.16	.031*	.01	4.45
Locus of control - internal	-1.95	0.65	-.12	-2.98	.003**	.01	8.81
Locus of control - external	1.39	0.54	.10	2.58	.010*	.01	6.79
**Depersonalization**							
Sense of coherence	-0.35	0.04	-.42	-8.32	< .001**	.10	69.74
Resilience	0.01	0.03	.01	0.26	.797	.00	0.09
Locus of control - internal	-0.31	0.43	-.03	-0.71	.479	.00	0.48
Locus of control - external	0.02	0.36	.00	0.06	.956	.00	0.01
**Personal accomplishment**							
Sense of coherence	0.24	0.05	.23	4.70	< .001**	.03	21.02
Resilience	0.28	0.04	.34	7.84	< .001**	.08	61.62
Locus of control - internal	1.20	0.53	.10	2.27	.024*	.01	5.15
Locus of control - external	0.88	0.44	.09	2.01	.045*	.01	4.08

Note. * $p < .05$; ** $p < .01$. The columns reporting ΔR^2 and ΔF refer to hierarchical regression analyses in which each variable was included in the last step. *p*-values of the beta-weights and ΔF are equal and hence not reported twice.
